# What is meant by patient-centredness being value-based?

**DOI:** 10.3109/02813432.2013.848543

**Published:** 2013-12

**Authors:** Charlotte Hedberg, Niels Lynøe

**Affiliations:** ^1^Centre for Family Medicine, Karolinska Institutet, Stockholm, Sweden; ^2^Centre for Healthcare Ethics, Karolinska Institutet, Stockholm, Sweden

**Keywords:** Autonomy, communication skill, general practice, patient-centredness, respectful encounters, Sweden, value-based medicine

## Abstract

**Objective:**

To examine whether it is possible to further specify what is meant when we maintain that patient-centredness as a communication skill is a value-based clinical procedure.

**Design and main outcome measures:**

Since a core element in patient-centredness is associated with patients feeling respected, a study regarding encounters where patients felt respected was analysed.

**Results:**

Similarities were found between the core elements of patient-centredness in terms of inviting, listening, and summarizing, and patients feeling respected in terms of listening, having their questions answered, and believing in what they tell their GP.

**Conclusion:**

Even though what is respected cannot be specified, the authors’ analysis indicates that feeling respected is frequently and strongly associated with encounters reflecting core aspects of patient-centredness. In this sense, patient-centredness might be considered value-based. Future research might shed light on what is actually respected: is it the patient's autonomy, integrity, dignity, or honour?

- Clinical procedures are considered value-based if patients feel respected and accordingly accept the procedures.
- Feeling respected is associated with health care providers who listen, answer the patient's questions, and believe in the patients, all being aspects similar to patient-centredness.
- Future research might elucidate what is actually respected: the patients’ autonomy, integrity, dignity, or honour?

## Introduction

Medical methods are supposed to be evidence-based before being implemented, but an evidence-based method can only be implemented if it is also accepted by the patient. In other words, a medical method is also supposed to be value-based [1]. If an evidence-based method is not value-based, its implementation might prove difficult and counterproductive [2]. This is also the case regarding communication skills assessed as medical methods [3]. Patient-centredness is a typical instance of communication skill. The question is whether or not patient-centredness is value-based and what being value-based implies.

Depending on how patient-centredness is defined and adherence to treatment outcome is measured, at least some aspects of the method seem to be evidence-based [3,4]. In one sense, patient-centredness is also value-based: the method is accepted and even appreciated by both patients and physicians [3]. But this is only a minimum demand for classifying a procedure as value-based. The question is whether we can further qualify what we mean by saying that such a method is value-based.

## Specifying value-based

To answer this question, we suggest examining whether the method results in patients feeling respected, which has been identified as a core element of patient-centredness [4]. In a recent study we identified a top-five list of specific encounter items associated with long-term sick-listed patients feeling respected [5]. According to the list, the feeling of being respected was most frequent and strongly associated with encounters where the health-care provider:

- listened to the patient,
- answered the patient's questions,
- believed in what the patient said,
- was competent,
- was committed.

Nevertheless, we would like to examine whether there are similarities between these specific top five encounters associated with patients feeling respected and encounters associated with patient-centredness.

## Discussion

There are different versions of patient-centredness and the associated communication skills [4], but it is commonly stressed that the health care provider should “invite, listen and summarize” [6]. The physician invites the patient to say why he or she is seeking health care – indicating that the physician is *interested* and *committed*. The next step involves the physician *listening* carefully without interrupting the patient for the first two or three minutes. While listening, the physician might focus on the patient's ideas, concerns, and expectations – and, accordingly, the physician will be in a better position to *answer the patient's questions* and also ask relevant questions. The third step is for the physician to summarize what the patient has said (using the patient's own words) – indicating that the physician listens, understands the patient's concern, and *believes* what the patient said. A physician who is committed, who listens without interrupting the patient, understands, and is prepared to answer the patient's questions (and avoid answering issues the patient did not ask) and, finally, take seriously what the patient says, will probably also be rated a nice and competent doctor.

As can be seen, encounters associated with patients feeling respected allow us to suggest that this version of patient-centredness is value-based. Actually, we might argue that the method is value-based in a more qualified manner, at least compared with being just accepted or appreciated. Future research might shed light on what is actually respected: is it the patient's autonomy, integrity, dignity, or honour [7]? Specifying what kinds of ethical principles or aspects are involved might be considered as a further qualification of what value-based means. But currently it seems good enough to know that the patient-centred method is value-based in a fairly qualified manner.
